# Centering Equity in the Nation's Weather, Water, and Climate Services

**DOI:** 10.1089/env.2022.0048

**Published:** 2024-02-07

**Authors:** Aradhna Tripati, Marshall Shepherd, Vernon Morris, Karen Andrade, Kyle Powys Whyte, Dominique M. David-Chavez, Justin Hosbey, Joseph E. Trujillo-Falcón, Brandon Hunter, Deanna Hence, DaNa Carlis, Vankita Brown, William L. Parker, Andrew Geller, Alex Reich, Mary Glackin

**Affiliations:** Dr. Aradhna Tripati is Professor at Department of Atmospheric and Oceanic Sciences, Center for Diverse Leadership in Science, Institute of the Environment and Sustainability, UCLA, Los Angeles, California, USA; Department of Earth, Planetary, and Space Sciences, American Indian Studies Center, UCLA, Los Angeles, California, USA.; Prof. Marshall Shepherd is Professor at Department of Geography, University of Georgia, Athens, Georgia, USA.; Prof. Vernon Morris is Professor at New College of School of Mathematical and Natural Sciences in the New College of New College for Interdisciplinary Arts and Sciences, Arizona State University, Tempe, Arizona, USA.; Dr. Karen Andrade is a Fellow at the Center for Diverse Leadership in Science, UCLA, Los Angeles, California, USA.; Dr. Kyle Powys Whyte is Professor at School for Environment and Sustainability, University of Michigan, Ann Arbor, Michigan, USA.; Prof. Dominique M. David-Chavez is an Assistant Professor at Department of Forest and Rangeland Stewardship, Colorado State University, Fort Collins, Colorado, USA.; Dr. Justin Hosbey is an Assistant Professor of City and Regional Planning at UC Berkeley, Berkeley, California, USA.; Mr. Joseph E. Trujillo-Falcón is a Graduate Research Assistant at the Cooperative Institute for Severe and High-Impact Weather Research and Operations, NOAA National Severe Storms Laboratory, Norman, Oklahoma, USA.; Dr. Brandon Hunter is a Fellow at the Center for Diverse Leadership in Science, UCLA, Los Angeles, California, USA; Postdoctoral Scholar at the Department of Earth and Environmental Engineering, Columbia University, New York City, New York, USA; Center for Rural Enterprise and Environmental Justice, Lowndes County, Alabama, USA.; Prof. Deanna Hence is an Assistant Professor at Department of Atmospheric Sciences, University of Illinois at Urbana-Champaign, Urbana, Illinois, USA.; Dr. DaNa Carlis is the Director of the National Severe Storms Laboratory, National Oceanic and Atmospheric Administration, Norman, Oklahoma, USA, USA.; Dr. Vankita Brown is a Senior Advisor for Equity at the National Oceanic and Atmospheric Administration, and a Research Social Scientist at the National Weather Service, Silver Spring, Maryland, USA.; Dr. William L. Parker is the Meteorologist in Charge at National Weather Service, National Oceanic and Atmospheric Administration, Jackson, Mississippi, USA.; Dr. Andrew Geller is the Senior Scientist and Executive Lead for Lead (Pb) and Environmental Justice Research at Office of Research and Development, U.S. Environmental Protection Agency, Research Triangle Park, North Carolina, USA.; Mr. Alex Reich is a Program Officer at the National Academies of Sciences, Engineering, and Medicine, Washington, District of Columbia, USA.; Dr. Mary Glackin was the Senior Vice President of Science and Forecast Operations at The Weather Company, IBM, Andover, Massachusetts, USA.

**Keywords:** Justice40, climate justice, weather, water, environmental justice, climate change

## Abstract

Water, weather, and climate affect everyone. However, their impacts on various communities can be very different based on who has access to essential services and environmental knowledge. Structural discrimination, including racism and other forms of privileging and exclusion, affects people's lives and health, with ripples across all sectors of society. In the United States, the need to equitably provide weather, water, and climate services is uplifted by the Justice40 Initiative (Executive Order 14008), which mandates 40% of the benefits of certain federal climate and clean energy investments flow to disadvantaged communities. To effectively provide such services while centering equity, systemic reform is required. Reform is imperative given increasing weather-related disasters, public health impacts of climate change, and disparities in infrastructure, vulnerabilities, and outcomes. It is imperative that those with positional authority and resources manifest responsibility through (1) recognition, inclusion, and prioritization of community expertise; (2) the development of a stronger and more representative and equitable workforce; (3) communication about climate risk in equitable, relevant, timely, and culturally responsive ways; and (4) the development and implementation of new models of relationships between communities and the academic sector.

## INTRODUCTION

In an interview at COP26 (26th UN Climate Change Conference of the Parties), when asked what climate change looked and felt like, Brianna Fruean, a young Pacific Islander who advocates for climate justice on behalf of her fellow Samoans, described the smell of thick mud she had to wade through and shovel out of houses after a flood.^1^ The multiple human dimensions of climate change are inescapable. *Anthropogenic climate change* is the disruptive response of our planet's weather and water cycles to human activity and development—largely emissions and land surface changes—that directly and indirectly affect energy balance in the Earth System.

Yet, what that definition does not name are the tangible and inequitably distributed consequences of this anthropogenic problem. *Climate injustice* refers to the role of structural discrimination in saddling communities of color and low-income communities with disproportionately high burdens of the harmful risks and impacts of climate change.^2^ In the United States and around the world, when climate disasters occur—whether extreme weather leading to floods, tornadoes, hurricanes, fires, or droughts—black, Indigenous, and other people of color (BIPOC) and frontline communities are disproportionately vulnerable to environmental and climate injustices.^3–10^

Structural racism and segregation are intrinsically linked to this vulnerability.^11^ Although no one is insulated from the climate crisis, BIPOC communities are most at risk. As equity gaps increase, skewed vulnerabilities and repeated disasters can destabilize families and communities for generations. For example, high concentrations of BIPOC communities live in flood-prone and/or water-poor areas.^12^^,^^13^ Racist U.S. Federal urban planning policies, including redlining, have resulted in a lack of trees and parks in areas of BIPOC communities, contributing to urban heat islands and higher mortality rates during heat waves.^14^^,^^15^

The nation's weather, water, and climate services, such as disaster risk management, community outreach, water resource assessment, swift and accurate information dissemination, hazard monitoring, education, workforce preparation, and more, play a key role in communities' well-being. Yet BIPOC communities often experience delays in response to climate emergencies and receive fewer resources for recovery,^4^^,^^16^^,^^17^ which can cascade into disasters of greater and longer magnitude, and intersect with other crises. For example, in Louisiana's “Cancer Alley,” delayed emergency response in the wake of Hurricane Ida further increased residents' risk of exposure to carcinogenic materials and increased transmission of coronavirus through seeking the same limited shelter accommodations.^18^

In the southeast United States, the National Weather Service (NWS) radar networks leave wide swaths of black communities without radar coverage.^19^ During and after Hurricane Katrina and other events, inequitable and racist policies and practices associated with risk management, service management, disaster response, and redevelopment contributed to reduced community resiliency and entrenching of health and wealth disparities through the loss of home, family, networks, and educational access and reform.^10^ Loss of relationship to place due to migration and forced displacement can cascade into a loss of culture, sovereignty (particularly for Indigenous communities), and autonomy, and into threats to civil liberties.^1^^,^^20^

Another dimension of this problem is that there is little representation from BIPOC communities in the geosciences.^21–23^ Less than 10% of National Oceanic and Atmospheric Administration (NOAA's) senior executive service members or NWS employees are from BIPOC communities, and 91% of NWS meteorologists and hydrologists are white based on March 2022 data from the NWS Equal Opportunity and Diversity Management Division. In contrast, over 42% of the U.S. population is nonwhite.^24^

This inadequate representation of BIPOC scientists means there is a lack of workers who understand the issues most relevant to BIPOC communities, which cascades into the development of inequitable policies, services, outreach, planning, and resource deployments that devalue and deprioritize the needs of BIPOC communities.^25–27^ These concerns are further heightened given the strengths of Indigenous and local knowledges for climate adaptation and resilience, and for safeguarding biodiversity.^28^ Both the Intergovermental Panel on Climate Change^29^ and the White House^30^ have affirmed that Indigenous peoples hold diverse Science, Technology, Engineering, and Mathematics (STEM) knowledges that can guide natural resources stewardship, water management, fire management, soil and water health, disaster response, and decision making.

It is critical to center equity if we are going to address intersecting climate, environmental, and social justice crises. Although these topics and related issues have been highlighted for decades, there has been insufficient action. In light of Executive Order 14008 and the Justice40 Initiative,^31^^,^^32^ this comment is intended as a resource and call to action for the services and science community.

## “THE FIERCE URGENCY OF NOW”

Given the current and future importance of the nation's weather, water, and climate services to the well-being of communities, it is imperative to apply an equity and justice framework to ensure these services serve everyone. The moral and public health imperatives of the climate crisis, and the continued human, social, and environmental costs of inaction, drive, to paraphrase Dr. Martin Luther King, Jr.,^33^ a “fierce urgency of now” around equitable and just action. We have a mandate to accelerate society's response to climate change, while simultaneously moving beyond good intentions and toward action.

The nation's services have an essential role to play in dismantling discriminatory structures and the building of new systems, informed by community knowledge. Without urgent and targeted action in response to the accelerating rate of climate change and extreme weather events, we will continue to see a widening of disparities, and we will continue to see inadequate, unequal, and unjust responses. The *No Time for Silence* call to action^34^ cast down a gauntlet for change and laid out a road map to move beyond recognizing the injustices, to taking specific actions, and has been endorsed by professional geoscience societies and departments representing >60,000 geoscientists.

## CENTERING EQUITY, NOT JUST DIVERSITY OR INCLUSION

The nation's weather, water, and climate services need equity-minded leaders who have resources, positional authority, and institutional agency to heed and respond to multiple calls for change.^34–37^ Correcting the effects of systemic discrimination requires changing cultures and sharing power, not solely increasing diversity that often results in bringing BIPOC individuals into hostile environments. Equity must be built in from the outset by identifying clear indicators and metrics with which to evaluate justice and equity outcomes^38–40^ (i.e., who will benefit and how, who will be included in decision making).

Environmental justice efforts must go beyond identifying disproportionate impacts, and directly engage with why certain communities were discriminated against.^41^ Equity in and of itself cannot be adequately addressed without recognizing and addressing systemic racism as a root cause of environmental inequities, and go beyond colorblind ideological frameworks and explicitly address institutionalized racism.^42^

Smart and intentional action planning can move levers of change in transformative ways. If the issues are taken to heart by forward-thinking leaders across the policy-making, corporate, academic, philanthropic, and nonprofit sectors, major steps can be taken to address these issues in the geosciences in the next decade. Leaders can drive institutional change now by modifying practices; bylaws; and local, state, and federal policies, to support more inclusive, equitable, and just organizations.

## ACTIONS TO ADVANCE EQUITY IN THE NATION'S WEATHER, WATER, AND CLIMATE SERVICES

Intentional and deliberate progress toward an equity framework will require leaders to prioritize four action areas: (1) recognizing and prioritizing community expertise; (2) building a stronger and more equitable workforce; (3) communicating about climate risk in equitable, timely, and culturally responsive ways; and (4) developing new models of relationship between communities and academia. Our goal is to identify current shortcomings and propose strategies to improve implementation.

### Recognizing and prioritizing community expertise

Communities need to be key partners in how scientists and policy-makers respond and adapt to weather, water, and climate issues, with a seat at the table.^38^^,^^43^ A close and authentic relationship between communities and services will facilitate effective and sustainable solutions that are culturally sensitive, timely, and relevant. Authenticity requires from the outset a sustained commitment to relational accountability and an in-person approach grounded in equity; justice; reciprocity; mutual trust; respect; and sharing of information, resources, and power.

Effective partnerships should respect the realities of communities, with a social, historical, and present-day trauma-informed context. Services and justice in BIPOC communities have been adversely impacted by institutional barriers and generations of disempowerment, which continue to limit all aspects of physical, mental, ecological, economic, and social health, as well as cultural life and community resilience. Research and engagement with communities need to be reimagined given that many BIPOC individuals, families, and communities—including people who are immigrants or undocumented—have significant reasons to distrust government or higher education entities,^3^^,^^44^ with a desire to avoid continuation of exploitation. This presents a difficult, yet not insurmountable challenge.

Although Western STEM and policy institutions are increasingly recognizing these exploitative outcomes and pledged to be more equitable and inclusive, much work is needed. Support is needed for communities to have the space, time, and resources to organize the involvement of their knowledge keepers, scientists, and knowledge systems in science and policy. This includes crafting inclusion in ways that reflect what it takes for communities to mobilize.

Institutions need to recognize the diverse forms of knowledge that communities may choose to invoke in particular circumstances. Indigenous people have used traditional knowledge systems to not only track environmental change over time, but also to take action to address climate change through efforts such as community-based planning for resettlement, the rebuilding of institutions, and actions to engage in self-determination and self-governance.^40^ In island communities, local and Indigenous knowledge systems serve critical roles in climate resilience, including by providing food security and architectural integrity in the face of increasing hurricane activity.^45–47^

Communities need updated time-relevant information on potential risks and available services to effectively plan and manage impacts, and should be included, nonexploitatively, and in ways that build community capacity, in determining what those services are. Community-based assessments are effective in determining what services are available, what is needed, and how they should be provided.

Direct payment for community member service on boards and committees are places where relationships can start to form and become long standing, in the same way that scientists' and policy-makers' salaries empower them to serve. Support is needed to ensure there is continuity of relationship, resources to complete the work, and a focus on building community capacity and centering community priorities.

In addition to providing resources, entities that provide services must update their processes to be more supportive of communities. They must share updates and results through briefings and communications to community members, and create agreements on governance and who holds authority throughout processes (e.g., establishing questions, collecting data, accessing findings, receiving credit, determining intellectual property rights, and guaranteeing meaningful outputs for communities).

Transparency and accountability mechanisms should be incorporated throughout, to ensure there are opportunities for determining what constitutes healthy and ethical interactions, and shifting course. A central goal should be rebuilding practices, rules, and organizational cultures so that communities are involved nonexploitatively from the outset. For services, this includes planning, education, research, operations, communications, and infrastructure development.

### Building a stronger and more equitable workforce

Equity indicators provide evidence of high rates of discrimination in geoscience training and workforce settings.^37^^,^^48–51^ Exclusion from educational opportunities, workforce rights, policy development, and leadership and decision-making roles perpetuates systemic racism, and ultimately protects the exclusionary nature of the status quo. This cycle is fundamental to the entrenchment of systemic racism in the academic and nonacademic workforce, and in individual institutions and organizations. As the climate crisis grows, current deficits in diversity and retention in the workforce and academy will continue to impede the development of effective and relevant weather, water, and climate services, priorities, and policies.

#### Equity in the workforce

Professionally, the representation of people of color in the federal STEM workforce lags behind the civilian workforce, which in turn significantly lags behind the nation's demographic profile.^20,47^ In the atmospheric science and meteorological workforce, there are ∼96,000 people working as postsecondary teachers, faculty, and instructors, yet fewer than 10 black full professors in atmospheric sciences and meteorology, and less than 15 in tenure-track positions.^20,47^ The private sector represents the fastest segment of growth in the weather, water, and climate enterprise and also has low levels of diversity.^23^^,^^52^

Thus there is a major need for representation in the nonacademic and academic workforce and in leadership that reflects the nation's demographics. Some companies work to directly recruit in and provide scholarship support to BIPOC students, minority-serving institutions (MSIs), and/or to programs for diverse workforce development at colleges and universities. Others use an equity lens to evaluate human resource policies on hiring and assessment, drawing on rubrics, and to evaluate whether applicants have meaningful engagement and experience with equity work.

Organizations and companies serious about centering equity and justice work should affirm their rationale for making these and other changes (e.g., to better the institution, to improve services, and/or to correct past unfair practices) and collaborate with community experts, whom they should recruit as employees, advisory board members, and consultants. In addition to necessitating the involvement of community experts, initiatives benefit from federal oversight to ensure data and programs are reliable and adherent to standards.

A recent RAND Corporation collaboration focused on reducing vulnerability while retaining resilience and involved company scientists partnering on a community-based participatory research project to catalog chemicals in maritime industrial areas and the risk of release in severe weather events. On issues that matter to impacted communities, companies and organizations should support advocacy to policy-makers and translate findings into action.

Recognizing that centering equity is challenging, strategies and programs should be evaluated to determine whether they are “easy,” or equity-avoidant strategies that are economically expedient, or whether they are in fact meaningful. Planning must address both immediate needs and long-term solutions, and consider cumulative long-term impacts on communities. There are opportunities to draw on principles of Indigeneity and focus on integrative life cycle analyses, which address water, climate, ecological, health, and equity impacts over multiple timescales, instead of only immediate costs.

#### Equity in education and training

Youth and students are future planners and climate leaders. From curiosity in the geosciences and STEM, a passion for addressing climate injustice and community engagement, to being a key vector for scientific innovation,^4^^,^^53^ BIPOC youth and students are desperately necessary to tackle the climate crisis. There are opportunities to listen and to include them in designing to build their own capacity, and the capacity of their communities, in ways that are forward looking. There is a pressing need for mentorship from people who share values with and believe in them and are grounded in strengths-based, not deficit-based, models, and a need for diverse pathways to scientific and policy leadership positions.

However, the geosciences remain persistently segregated due to issues with culture.^21,37,48,51^ In 2018, only 207 black students from a total pool of 7431 students graduated with undergraduate degrees in earth, atmospheric, and ocean sciences, and this number had barely doubled over the preceding decade.^21,23,30,50^ The institutions making significant impact are few.^23,49,50^^,^^54^ Given the culture of geoscience, trainees and more senior participants from marginalized groups continue to face hostility, isolation, and other barriers.^55–59^

Such experiences impact retention and recruitment by causing continual damage to BIPOC people's mental and physical health.^60^ Higher education needs to replace traditional models that demand that individuals persist in inhospitable or hostile environments, or leave, with redesigned environments and revised policies and practices that make spaces, partnerships, and institutions more welcoming and equitable, so all can thrive. Changes in practices and policies in the academy are also needed, including around admissions, hiring,^61^ tenure, and promotion, retention, paper reviews,^62^ funding,^63^ awards,^64^^,^^65^ social norms, and other aspects of traditional academia. Such changes will have a cascade of downstream effects, given the role of higher education in transmitting culture.

Geosciences education and research need to focus on redressing colonial relationships that are strongly expressed in science.^66^^,^^67^ Some models show that change is possible.^37,54^ MSIs have made headway in equity and access issues that impact undergraduate and graduate education. Howard University in partnership with NOAA produced 60% of all African American atmospheric sciences PhDs in the past decade with 98% in the workforce, whereas top 10 programs produced fewer than 10 in the past 25 years.^23,36,50,54^

Other examples of effective geoscience programs include NOAA's José E. Serrano Education Partnership Program with MSIs,^68^^,^^69^ responsible for training graduates who have become 37% of the NOAA minority science hires from 2000 to 2005; the Center for Diverse Leadership in Science at UCLA,^70–72^ which takes a human-centered relational approach grounded in equity, and works to build power in marginalized people, groups, and communities, while taking an interdisciplinary, intergenerational, and ecological approach, and has supported >200 fellows including students, community practitioners, and faculty fellows, and is effective at diversifying the geosciences workforce; and the Significant Opportunities in Atmospheric Research and Science program at University Corporation for Atmospheric Research that through effective mentored research experiences is significantly broadening graduate school participation of historically under-represented groups in the geosciences.^67^^,^^73^

Major increases in investment for effective strategies have recently been proposed to broaden participation in STEM and advance systemic change.^68^^,^^74^ Such an approach could be targeted to develop equity-driven centers focused on basic and applied climate, environment, and sustainability research, education, and partnerships.

Another key area for higher education reform relates to the development of curricula on climate and environmental justice and ethics, and how they are related to the geosciences. Topics could include resource extraction, land dispossession, settler colonialism, structural racism, pollution, climate change, extreme weather events, and their inter-relationships. Within these materials, emphasizing traditional knowledge systems and sustainability in Western and Indigenous contexts will enable this country's next generation of students and leaders to have broader critical awareness.

### Communicating climate risk: equitable, timely, and culturally responsive

The nation's weather, water, and climate services have the responsibility of providing equitable access to accurate and up-to-date information and resources for all, regardless of language or documentation status, with equitable rules and mechanisms for distributing resources. Greater federal investment is needed to assist communities with climate preparedness and resiliency planning, and to support agencies producing knowledge around equitable and culturally responsive provision of services.

Government agencies must provide accessible tools and products; to ensure community confidence in these resources, they must also support community expertise and ambassadorship in the design and deployment of climate services, and offer opportunities for community evaluation and feedback. It is critical that agency workforces listen to community leaders and build relationships between different and diverse communities.

Risk and crisis communication also necessitate connection and partnership with communities. For example, communities may speak different languages, or dialects of a language, which can impact the ability of community members to fully participate—including being reached with emergency alerts—unless paid translators are provided.^4^

Language accessibility cannot be sustained by communities alone: government agencies should fund and develop multilingual centers that not only produce effective translations for life-saving hazards, but also guarantee connections with community partners. Key documents representing community voices and leadership show that current efforts are not enough, such as was highlighted in recent recommendations on environmental and climate justice by the White House Environmental Justice Advisory Council.^32^

Poor infrastructure and information availability are major impediments that lead to disproportionate impacts from environmental disasters in underserved communities. To ensure equitable services, harm reduction, mitigation, and adaptation need to be emphasized, with granular information at the level of neighborhoods, localities, communities, and tribal nations. Verification of critical services should be expanded to understand who is impacted, what the impacts are, what languages are relevant, how information is received, and who benefits from existing and new resources. Given fragmentation of existing services across different agencies, new interagency coordination is needed among services, with equity centered from the outset. Additional capacity is severely needed.

The United States Geological Survey (USGS) Climate Adaptation Science Centers (CASCs), a partnership-driven program wherein scientists collaborate with natural and cultural resource managers to combat climate change, and the NOAA Regional Integrated Sciences and Assessments (RISAs) programs, which supports applied and co-developed research partnerships with communities to combat climate change, are examples. USGS CASCs and NOAA RISAs emphasize transferring science and data to local communities, and additional regional and local weather services capacity such as these models are critical to effectively addressing inequities.

Collection and assessment of demographic data are needed to understand who becomes ill or dies because of extreme events, climate change, and pollution; what health and warning information they had; and whether they had access to harm mitigation resources (e.g., local cooling centers during extreme heat). When collecting demographic data, strategies in counting marginalized populations should be re-evaluated.^6^^,^^75^ Overall, a joint effort of physical, social, and health scientists is needed to study and understand disparate and inequitable climate change vulnerabilities and impacts.^70^^,^^76^ National and regional climate assessments should more thoroughly present the unequally distributed impacts on different demographics.

### Alternative models of relationship: communities and the academic sector

Educational institutions should revisit what it means to be a geoscientist, what valued education and research look like, and the many roles geoscientists can have with communities and employers. Institutions have the opportunity to reframe and build a professional culture in which working with local communities and a science career are synergistic, not exclusive as traditionally framed in STEM, and can provide deep long-term investments to build and sustain community integration. Departments and universities should support training and practice in applied opportunities, including community-based work.

One example can be found in Science Shops, crafted in response to concerns raised by the popular education and participatory action research movements in the global south, which coordinates community-engaged research that centers the needs of community-based organizations that brings in university-based scientists and students.^71^^,^^77^ In American universities, it has demonstrated both its ability to channel community voice into research agendas and potential for catalyzing institutional culture change.^78^ Programs such as American Geophysical Union's Thriving Earth Exchange train volunteer scientists to work in authentic ways in support of community environmental projects.^79^

Highlighting a breadth of career options and role models, and fostering multiple career pathways, would help future scientists and science fields as a whole. Institutions need to understand the sacrifices most programs ask of students for their training (e.g., separation from home communities, support structures, and relationship to place), which can cause harm to people from structurally excluded groups, particularly Indigenous students. Much can be learned from nontraditional models utilized by academic experts, including extension programs, such as used in the department of environmental science at the University of Arizona by Prof. Karletta Chief,^80^ and other frameworks such as that developed by Prof. Bryan Brayboy at Arizona State University centered on empowering, engaging, and enhancing communities.^81–83^

Finally, funding opportunities should require that research is framed using an equity lens, with explicitly developed action plans to allow for self-determined rather than exploitative community engagement.^58^ For community-based work, research planning should include discussions of listening and engagement sessions with partners, and review processes should include information on protocols and consents, in the model of institutional review board protocols and similar structures in many tribal organizations. Social vulnerabilities need to be explicitly incorporated in resilience research, policy, and planning.^70^

## CONCLUSION

The necessary work of moving from good intentions to sustained action lies ahead. Although past efforts have often been piecemeal, incremental, and not maintainable over time, the intersections of climate change with inequality demand that equity, as well as justice and inclusion, be centered across the board. All status-quo processes, systems, and procedures must be examined and challenged. Institutional leaders must adopt an equity-centered mindset to facilitate the implementation of effective, inclusive, and sustainable solutions that will save lives. The necessary changes are reflected in recent calls to change policy to achieve systemic reform, to share power and information, and to ensure equitable access to networks and resources.

Leaders with positional authority and resources must manifest responsibility through action in the four areas already described: (1) recognizing, integrating, and prioritizing community expertise; (2) building a stronger and more equitable workforce; (3) communicating about climate risk in equitable, relevant, timely, and culturally responsive ways; and (4) developing new models of relationship between communities and the academic sector. Collectively, these actions will help to ensure there is equity in the nation's weather, climate, and water services, for current and future generations.

## Notes

^1^
National Public Radio. 2021. <https://www.npr.org/transcripts/1054655933>. (Last accessed on October 10, 2022).

^2^
Marshall Shepherd. “The Curious Relationship Between COVID-19 Lockdowns and Urban Heat Islands.” *Geophysical Research Letters* 49 (2022): e2022GL098198.

^3^
Angel Hsu, Glenn Sheriff, Tirthankar Chakraborty, and Diego Manya. “Disproportionate Exposure to Urban Heat Island Intensity Across Major US Cities.” *Nature Communications* 12 (2021): 1–11.

^4^
Joseph E. Trujillo-Falcón, Orlando Bermúdez, Krizia Negrón-Hernández, John Lipski, Elizabeth Leitman, and Kodi Berry. “Hazardous Weather Communication En Español: Challenges, Current Resources, and Future Practices.” *Bulletin of the American Meteorological Society* 102 (2021): E765–E773.

^5^
Neil Debbage. “Multiscalar Spatial Analysis of Urban Flood Risk and Environmental Justice in the Charlanta Megaregion, USA.” *Anthropocene* 28 (2019): 100226.

^6^
Robert D. Bullard. *Dumping in Dixie: Race, Class, and Environmental Quality*. (Routledge, 2018).

^7^
Michael Méndez, Genevieve Flores-Haro, and Lucas Zucker. “The (In)visible Victims of Disaster: Understanding the Vulnerability of Undocumented Latino/a and Indigenous Immigrants.” *Geoforum* 116 (2020): 50–62.

^8^
Robert Bullard. “Addressing Environmental Racism.” *Journal of International Affairs* 73 (2019): 237–242.

^9^
Marshall Shepherd and K.C. Binita. “Climate Change and African Americans in the USA.” *Geography Compass* 9 (2015): 579–591.

^10^
Justin Hosbey. “Refusing Unliveable Destinies: Toward a Future for Black Life in New Orleans.” *Fire!!!* 5 (2018): 35–47.

^11^
Fayola Jacobs. “Black Feminism and Radical Planning: New Directions for Disaster Planning Research.” *Planning Theory* 18 (2019): 24–39.

^12^
Eliot M. Tretter and Melissa Adams. “The Privilege of Staying Dry: The Impact of Flooding and Racism on the Emergence of.” In: *Cities, Nature and Development: The Politics and Production of Urban Vulnerabilities.* (Routledge, 2012), 187.

^13^
Joel E. Correia. “Between Flood and Drought: Environmental Racism, Settler Waterscapes, and Indigenous Water Justice in South America's Chaco.” *Annals of the American Association of Geographers* 112 (2022): 1890–1910.

^14^
Jeremy S. Hoffman, Vivek Shandas, and Nicholas Pendleton. “The Effects of Historical Housing Policies on Resident Exposure to Intra-Urban Heat: A Study of 108 US Urban Areas.” *Climate* 8 (2020): 12.

^15^
Richard Rothstein. *The Color of Law*. (Liveright Publishing, 2017).

^16^
Carlos E. Rodríguez-Díaz. “Maria in Puerto Rico: Natural Disaster in a Colonial Archipelago.” *American Journal of Public Health* 108 (2018): 30–32.

^17^
Christopher Flavelle. “Why Does Disaster Aid Often Favor White People?” *New York Times* (2021).

^18^
Rachel Ramirez and Nicquel Terry Ellis. “What a Hurricane Means When You Live in Louisiana's ‘Cancer Alley’.” *CNN* (2021).

^19^
Marshall Shepherd. “Are Black and Rural Residents in the South More Vulnerable to Tornadoes Due to Radar Gaps?” *Forbes* (2021).

^20^
Julie Koppel Maldonado, Christine Shearer, Robin Bronen, Kristina Peterson, and Heather Lazrus. “The Impact of Climate Change on Tribal Communities in the US: Displacement, Relocation, and Human Rights.” In: *Climate Change and Indigenous Peoples in the United States*. (Springer, 2013), 93–106.

^21^
National Center for Science and Engineering Statistics (NCSES). *Women, Minorities, and Persons with Disabilities in Science and Engineering*. (National Science Foundation, 2021).

^22^
Suzanne Van Cooten. “Where Are the Indigenous Scientific Leaders? Examining the Participation of Native American/Alaska Natives in Weather and Water Academic Programs and the Federal Workforce.” *Bulletin of the American Meteorological Society* 95 (2014): 1725–1740.

^23^
Vernon Morris, Solomon Bililign, and Belay Demoz. “The Good, the Bad, and the Ugly: Sobering Statistics on Black Atmospheric Scientists in the Academy.” In: *101st American Meteorological Society Annual Meeting*. (AMS, 2021).

^24^
United States Census Bureau. *Census Bureau Releases Estimates of Undercount and Overcount in the 2020 Census*. (United States Census Bureau, 2022).

^25^
Kristin E. Henkel, John F. Dovidio, and Samuel L. Gaertner. “Institutional Discrimination, Individual Racism, and Hurricane Katrina.” *Analyses of Social Issues and Public Policy* 6 (2006): 99–124.

^26^
Catherine Coleman Flowers. *Waste: One Woman's Fight Against America's Dirty Secret*. (The New Press, 2020).

^27^
Jennie C. Stephens. *Diversifying Power: Why We Need Antiracist, Feminist Leadership on Climate and Energy*. (Island Press, 2020).

^28^
Álvaro Fernández-Llamazares, Dana Lepofsky, Ken Lertzman, Chelsey Geralda Armstrong, Eduardo S. Brondizio, Michael C. Gavin, Phil O'B. Lyver, George P. Nicholas, Pua'ala Pascua, Nicholas J. Reo, Victoria Reyes-García, Nancy J. Turner, Johanna Yletyinen, E.N. Anderson, William Balée, Joji Cariño, Dominique M. David-Chavez, Christopher P. Dunn, Stephen C. Garnett, Spencer Greening, Shain Jackson, Harriet Kuhnlein, Zsolt Molnár, Guillaume Odonne, Gunn-Britt Retter, William J. Ripple, László Sáfián, Abolfazl Sharifian Bahraman, Miquel Torrents-Ticó, and Mehana Blaich Vaughan “Scientists' Warning to Humanity on Threats to Indigenous and Local Knowledge Systems.” *Journal of Ethnobiology* 41 (2021): 144–169.

^29^
W. Neil Adger, Juan M. Pulhin, Jon Barnett, Geoffrey D. Dabelko, Grete K. Hovelsrud, Marc Levy, Ursula Oswald Spring, and Coleen H. Vogel, IPCC Climate. “Impacts, Adaptation, and Vulnerability.” *Part A: Global and Sectoral Aspects. Contribution of Working Group II to the Fifth Assessment Report of the Intergovernmental Panel on Climate Change* 1132 (2014).

^30^
Memorandum on Indigenous Traditional Ecological Knowledge and Federal Decision Making. 2021. <Whitehouse.gov>. (Last accessed on October 10, 2022).

^31^
Joseph Biden Jr. “Executive Order on Tackling the Climate Crisis at Home and Abroad.” Executive Order 14008, White House (2021).

^32^
White House Environmental Justice Advisory Council Final Recommendations: Justice40 Climate and Economic Justice Screening Tool & Executive Order 12898 Revisions (2021).

^33^
Martin Luther King Jr. “Speech at the March on Washington” (1963).

^34^
Vernon Morris, Lisa White, Jose Fuentes, Christopher Atchison, Wendy Smythe, Melissa Burt, Leticia Williams, Aradhna Tripati, Belay Demoz, and Roy Armstrong, “No Time For Silence: A Call to Action for an Anti-Racist Science Community from Geoscientists of Color: Listen, Act, Lead.” 2020. <https://notimeforsilence.org/>. (Last accessed on October 10, 2022).

^35^
Hendratta Ali. “Call for a Robust Anti-Racism Plan for the Geosciences.” 2020. <Change.org>. (Last accessed on October 10, 2022).

^36^
Hendratta N. Ali, Sarah L. Sheffield, Jennifer E. Bauer, Rocío P. Caballero-Gill, Nicole M. Gasparini, Julie Libarkin, Kalynda K. Gonzales, Jane Willenbring, Erika Amir-Lin, Julia Cisneros, Dipa Desai, Maitri Erwin, Elisabeth Gallant, Kiara Jeannelle Gomez, Benjamin A. Keisling, Robert Mahon, Erika Marín-Spiotta, Leiaka Welcome, and Blair Schneider. “An Actionable Anti-Racism Plan for Geoscience Organizations.” *Nature Communications* 12 (2021): 1–6.

^37^
Vernon R. Morris. “Combating Racism in the Geosciences: Reflections from a Black Professor.” *AGU Advances* 2 (2021): e2020AV000358.

^38^
Linda Shi, Eric Chu, Isabelle Anguelovski, Alexander Aylett, Jessica Debats, Kian Goh, Todd Schenk, Karen C. Seto, David Dodman, Debra Roberts, J. Timmons Roberts, and Stacy D. VanDeveer. “Roadmap Towards Justice in Urban Climate Adaptation Research.” *Nature Climate Change* 6 (2016): 131–137.

^39^
Heather Lazrus, Julie Maldonado, Paulette Blanchard, M. Kalani Souza, Bill Thomas, and Danial Wildcat. “Culture Change to Address Climate Change: Collaborations with Indigenous and Earth Sciences for More Just, Equitable, and Sustainable Responses to Our Climate Crisis.” *PLoS Climate* 1 (2022): e0000005.

^40^
Dominique M. David-Chavez and Michael C. Gavin. “A Global Assessment of Indigenous Community Engagement in Climate Research.” *Environmental Research Letters* 13 (2018): 123005.

^41^
David Schlosberg. “Theorising Environmental Justice: The Expanding Sphere of a Discourse.” *Environmental Politics* 22 (2013): 37–55.

^42^
J.L. Harrison. “Bureaucrats' Tacit Understandings and Social Movement Policy Implementation: Unpacking the Deviation of Agency Environmental Justice Programs from EJ Movement Priorities.” *Social Problems* 63 (2016): 534–553.

^43^
Isabelle Anguelovski, Linda Shi, Eric Chu, Daniel Gallagher, Kian Goh, Zachary Lamb, Kara Reeve, and Hannah Teicher. “Towards Critical Studies of Climate Adaptation Planning: Uncovering the Equity Impacts of Urban Land Use Planning.” (2016).

^44^
Elizabeth Fussell, Linda Delp, Kevin Riley, Sergio Chávez, and Abel Valenzuela Jr. “Implications of Social and Legal Status on Immigrants' Health in Disaster Zones.” *American Journal of Public Health* 108 (2018): 1617–1620.

^45^
Status of Tribes and Climate Change Working Group (STACCWG). “Status of Tribes and Climate Change Report,” Institute for Tribal Environmental Professionals, Northern Arizona University, Flagstaff, AZ. [Marks-Marino, D. (ed.)]. 2021. <http://nau.edu/stacc2021>. (Last accessed on October 10, 2022).

^46^
Dominique David-Chavez and Norma Ortiz. “Strengthening Climate Resilience in the Caribbean Through an Indigenous Community-Based Participatory Research Model.” *AGU Fall Meeting Abstracts* (2018).

^47^
UNESCO. *Mobilizing Indigenous and Local Knowledge Solutions: Addressing Climate Impacts and Vulnerabilities A Perspective from the Caribbean Region. [Workshop Report]*. (United Nations Educational, Scientific and Cultural Organization, 2020).

^48^
Rachel E. Bernard and Emily H.G. Cooperdock. “No Progress on Diversity in 40 Years.” *Nature Geoscience* 115 (2018): 292–295.

^49^
Vernon R. Morris and Talitha M. Washington. “The Role of Professional Societies in STEM Diversity.” *Not Am Math Soc* 65 (2017).

^50^
Rachel J. Beane, Eric Baer, Rowan Lockwood, R. Heather Macdonald, John R. McDaris, Vernon R. Morris, I. Joshua Villalobos, and Lisa D. White. “Uneven Increases in Racial Diversity of US Geoscience Undergraduates.” *Communications Earth & Environment* 2 (2021): 1–4.

^51^
Steve Boss. *Modeling the Demographic Dynamics of the Geosciences Workforce*. (MSU Colloqium, 2021) (slides in possession of lead author).

^52^
United States Bureau of Labor Statistics. <https://www.bls.gov/opub/mlr/2020/>. (Last accessed on October 10, 2022).

^53^
Bas Hofstra, Vivek V. Kulkarni, Sebastian Munoz-Najar Galvez, Bryan He, Dan Jurafsky, and Daniel A. McFarland. “The Diversity–Innovation Paradox in Science.” *Proceedings of the National Academy of Sciences United States of America* 117 (2020): 9284–9291.

^54^
Vernon R. Morris, Everette Joseph, Sonya Smith, and Tsann-wang Yu. “The Howard University Program in Atmospheric Sciences (HUPAS): A Program Exemplifying Diversity and Opportunity.” *Journal of Geoscience Education* 60 (2012): 45–53.

^55^
Barbara Flores, Patricia Tefft Cousin, and Esteban Diaz. “Transforming Deficit Myths About Learning, Language, and Culture.” *Language Arts* 68 (1991): 369–379.

^56^
Chester M. Pierce, Jean V. Carew, Diane Pierce-Gonzalez, and Deborah Wills. “An Experiment in Racism: TV Commercials.” *Education and Urban Society* 10 (1977): 61–87.

^57^
Daniel Solorzano, Miguel Ceja, and Tara Yosso. “Critical Race Theory, Racial Microaggressions, and Campus Racial Climate: The Experiences of African American College Students.” *Journal of Negro Education* (2000): 60–73.

^58^
Claude M. Steele and Joshua Aronson. “Stereotype Threat and the Intellectual Test Performance of African Americans.” *Journal of Personality and Social Psychology* 69 (1995): 797.

^59^
Asmeret Berhe. “Amplifying Black Voices: The Convenient Narratives That Perpetuate Racism. 2020. <https://www.springernature.com/gp/researchers/the-source/blog/blogposts-life-in-research/amplifying-black-voices-the-convenient-narratives-of-racism-pt1/18415774>. (Last accessed on October 10, 2022).

^60^
Juanita C. Limas, Linda C. Corcoran, Alexander N. Baker, Ana E. Cartaya, and Zoë J. Ayres. “The Impact of Research Culture on Mental Health & Diversity in STEM.” *Chemistry–A European Journal* (2022): e202102957.

^61^
Özlem Sensoy and Robin DiAngelo. ““We Are All for Diversity, But…”: How Faculty Hiring Committees Reproduce Whiteness and Practical Suggestions for How They Can Change.” *Harvard Educational Review* 87 (2017): 557–580.

^62^
Kyle Powys Whyte. *Systematic Discrimination in Peer Review: Some Reflections*. (Daily Nous, 2017).

^63^
Diamond Tachera. *Reframing Funding Strategies to Build Reciprocity*. (Eos, 2021), 102.

^64^
Mary Anne Holmes, LaToya Myles, and Blair Schneider. “Diversity and Equality in Honours and Awards Programs–Steps Towards a Fair Representation of Membership.” *Advances in Geosciences* 53 (2020): 41–51.

^65^
Olivier Pourret, Pallavi Anand, Sandra Arndt, Pieter Bots, Anthony Dosseto, Zimin Li, Johanna Marin Carbonne, Jennifer Middleton, Bryne Ngwenya, and Amy J.V. Riches. “Diversity, Equity, and Inclusion: Tackling Under-Representation and Recognition of Talents in Geochemistry and Cosmochemistry.” *Geochimica et Cosmochimica Acta* 310 (2021): 363–371.

^66^
Max Liboiron. “Decolonizing Geoscience Requires More Than Equity and Inclusion.” *Nature Geoscience* 14 (2021): 876–877.

^67^
Kathryn Yusoff. *A Billion Black Anthropocenes or None.* (University of Minnesota Press, 2018).

^68^
Larry Robinson, Jacqueline Rousseau, Delicia Mapp, Vernon Morris, and Meka Laster. “An Educational Partnership Program with Minority Serving Institutions: A Framework for Producing Minority Scientists in NOAA-Related Disciplines.” *Journal of Geoscience Education* 55 (2007): 486–492.

^69^
Vernon Morris, T.-W. Yu, E. Joseph, R. Armstrong, R. Fitzgerald, R. Karim, X-Z. Liang, and Q. Min. “THE NOAA CENTER FOR ATMOSPHERIC SCIENCES (NCAS) Programs and Achievements.” *Bulletin of the American Meteorological Society* 88 (2007): 141–145.

^70^
Aradhna Tripati. “Transforming Geoscience and Environmental Science: Advancing Systemic Change Through Faculty and Early Career Development to Support Retention and Improved Institutional Climates.” *AGU Fall Meeting Abstracts* (2019).

^71^
Aradhna Tripati, Vernon Morris, Catalina Martinez, and Kendall Moore. “Human-Centered, Relational Geoscience.” *AGU Fall Meeting Abstracts* (2019).

^72^
Aradhna Tripati, Shanna Shaked, Krystle Cobian, Sylvia Hurtado, Marilyn Raphael, Ashley Kruythoff, Steve Dunwoody, Ingrid Maradiaga, Al Courey, Kristina Larsen. “Transforming Geoscience: Changing the Culture and Widening the Pathways.” *AGU Fall Meeting Abstracts* (2018).

^73^
Rajul E. Pandya, Sandra Henderson, Richard A. Anthes, and Roberta M. Johnson. “BEST Practices for Broadening Participation in the Geosciences: Strategies from the UCAR Significant Opportunities in Atmospheric Research and Science (SOARS^®^) Program.” *Journal of Geoscience Education* 55 (2007): 500–506.

^74^
Freeman A. Hrabowski III and Peter H. Henderson. “Nothing Succeeds Like Success.” *Issues in Science and Technology* (2021).

^75^
Trujillo-Falcón, Joseph E., América R. Gaviria Pabón, Joseph T. Ripberger, Abby Bitterman, Jonathan B. Thornton, Makenzie J. Krocak, Searn R. Ernst, Estilita Cassiani Obeso, and John Lipski. “¿Aviso o Alerta? Developing Effective, Inclusive, and Consistent Watch and Warning Translations for U.S. Spanish Speakers.” *Bulletin of the American Meteorological Society* 103 (2022): E2791–E2803.

^76^
Susan L. Cutter, Jerry T. Mitchell, and Michael S. Scott. “Revealing the Vulnerability of People and Places: A Case Study of Georgetown County, South Carolina.” *Annals of the Association of American Geographers* 90 (2000): 713–737.

^77^
Michael Søgaard Jørgensen and Merete Hende. “Science Shops-a Concept for Community Based Learning.” In: *New Engineering Competencies-Changing the Paradigm*. (SEFI: European Association for Engineering Education, 2001).

^78^
Karen Andrade, Lara Cushing, and Ashton Wesner. “Science Shops and the US Research University: A Path for Community-Engaged Scholarship and Disruption of the Power Dynamics of Knowledge Production.” In: *Educating for Citizenship and Social Justice*. (Palgrave Macmillan, 2018), 149–165.

^79^
Veera Mitzner, Rajul Pandya, Natasha Udu-gama, Judit Ungvari, and Eleanor Robson. “Thriving Earth Exchange and Future Earth: Strengthening Transdisciplinary and Community Science Around the World.” In: *AGU Fall Meeting 2021*. (AGU, 2021).

^80^
Karletta Chief. “Emerging Voices of Tribal Perspectives in Water Resources.” *Journal of Contemporary Water Research & Education* 163 (2018): 1–15.

^81^
B.M.J. Brayboy and Megan Bang. “Societal Issues Facing Indigenous Education: Introduction.” *Handbook of Indigenous Education* (2019): 567–574.

^82^
Bryn McKinley Jones Brayboy. *A Study of Indigenous Boys and Men*. (RISE for Boys and Men of Color, 2017).

^83^
Jessica A. Solyom, Jeremiah A. Chin, Bryan McKinley, Jones Brayboy, Amber Poleviyuma, Sarah Abuwandi, Alexus Richmond, Amanda Tachine, Colin Ben, and Megan Bang. “Systems of Support: What Institutions of Higher Education Can Do for Indigenous Communities.” *Handbook of Indigenous Education* (2019): 605.

